# Immunoradiotherapy for NSCLC: mechanisms, clinical outcomes, and future directions

**DOI:** 10.1007/s12094-023-03337-9

**Published:** 2023-11-03

**Authors:** He Weishan, Zheng Donglin, Deng Guangmei, Liu Wenya, Wu Fasheng, Chen Jibing

**Affiliations:** 1 Graduate School, Guangxi University of Chinese Medicine, Nanning, Guangxi China; 2 Ruikang Hospital, Guangxi University of Chinese Medicine, Nanning, Guangxi China

**Keywords:** Radiotherapy, Immunotherapy, Immune checkpoint inhibitor, Immunoradiotherapy, Non-small-cell lung cancer

## Abstract

Non-small-cell lung cancer (NSCLC) has an extremely low 5-year survival rate, with the only effective treatment being immunoradiotherapy (iRT). Here, we review the progress of clinical research on iRT for non-small-cell lung cancer (NSCLC) over 2018–2023, as well as the future directions. We first discuss the synergistic mechanisms of iRT, reflected in three aspects: immune regulation of RT, RT-activated immune-related pathways, and RT-related immune sensitization. iRT may include either external-beam or stereotactic-body RT combined with either immune checkpoint inhibitors (e.g., immunoglobulins against immune programmed cell death (PD) 1/PD ligand 1 or CD8^+^ T lymphocyte antigen 4) or traditional Chinese medicine drugs. Regarding clinical effectiveness and safety, iRT increases overall and progression-free survival and tumor control rate among patients with NSCLC but without a considerable increase in toxicity risk. We finally discuss iRT challenges and future directions reported over 2018–2023.

## Introduction

Lung cancer is a malignancy with a high incidence rate and mortality: 2.2 million new lung cancer cases and 1.8 million deaths were reported worldwide in 2020 [1]. Non-small-cell lung cancer (NSCLC) accounts for 85% of all lung cancer cases, and most patients are diagnosed as having NSCLC at an advanced stage, which makes them ineligible for surgical resection [2]. As such, the 5-year survival rate of patients with stage IV remains at only 5% [3].

Here, we review the progress in immunoradiotherapy (iRT) for NSCLC over 2018–2023 and explore techniques for strengthening it further.

### Radiation therapy

Radiotherapy (RT) is an effective therapeutic strategy for NSCLC, particularly in NSCLC patients ineligible for surgery. Older adults and smokers, who are prone to respiratory disorders (e.g., chronic obstructive pulmonary disease) and heart disease, have the highest risk of lung cancer; moreover, a considerable proportion of patients with lung cancer can only tolerate noninvasive therapy [3]. Recent technological advancements have led to the development of safer, more effective RT techniques and dosages for patients with NSCLC [4]. Consequently, the 3-year relative survival rate of patients with NSCLC has increased from 25% over 2004–2006 to 38% over 2016–2018 [5]. However, regardless of the radiation and dose segmentation approach used, some patients may develop radioresistance, resulting in RT failure and local relapse. In these cases, only raising the radiation dose may not enhance survival benefits but result in undesirable effects and poor prognosis [6]. To overcome these limitations, developing a novel bio-optimization strategy for RT with an acceptable safety profile and sustained response is necessary [7].

### Immunotherapy

Immunotherapy is effective in treating various cancers, including NSCLC [8]. Cancer incidence and progression are associated with an individual’s immunity or immune monitoring [9]. Immune checkpoint inhibitors (ICIs), such as anti-CTLA-4-antibodies (CTLA-4) and programmed cell death (PD) 1/PD ligand 1 (PD-L1), have revolutionized immune-management of advanced NSCLC [10]. Currently, PD-1/PD-L1 inhibitors such as pembrolizumab and atezolizumab are recommended for first-line therapy in advanced NSCLC patients with high PD-L1 expression [11–14]. Despite the encouraging outcomes associated with ICIs, only a few patients have demonstrated an overall sustained response thus far; most patients tend to develop primary resistance through the tumor immunity avoidance mechanism [15]. In addition, patients demonstrating a primary response may develop secondary resistance to the ICIs [16]. Therefore, studies on preventing the development of primary and secondary responses to ICIs in patients with NSCLC are warranted.

**Fig. 1:**
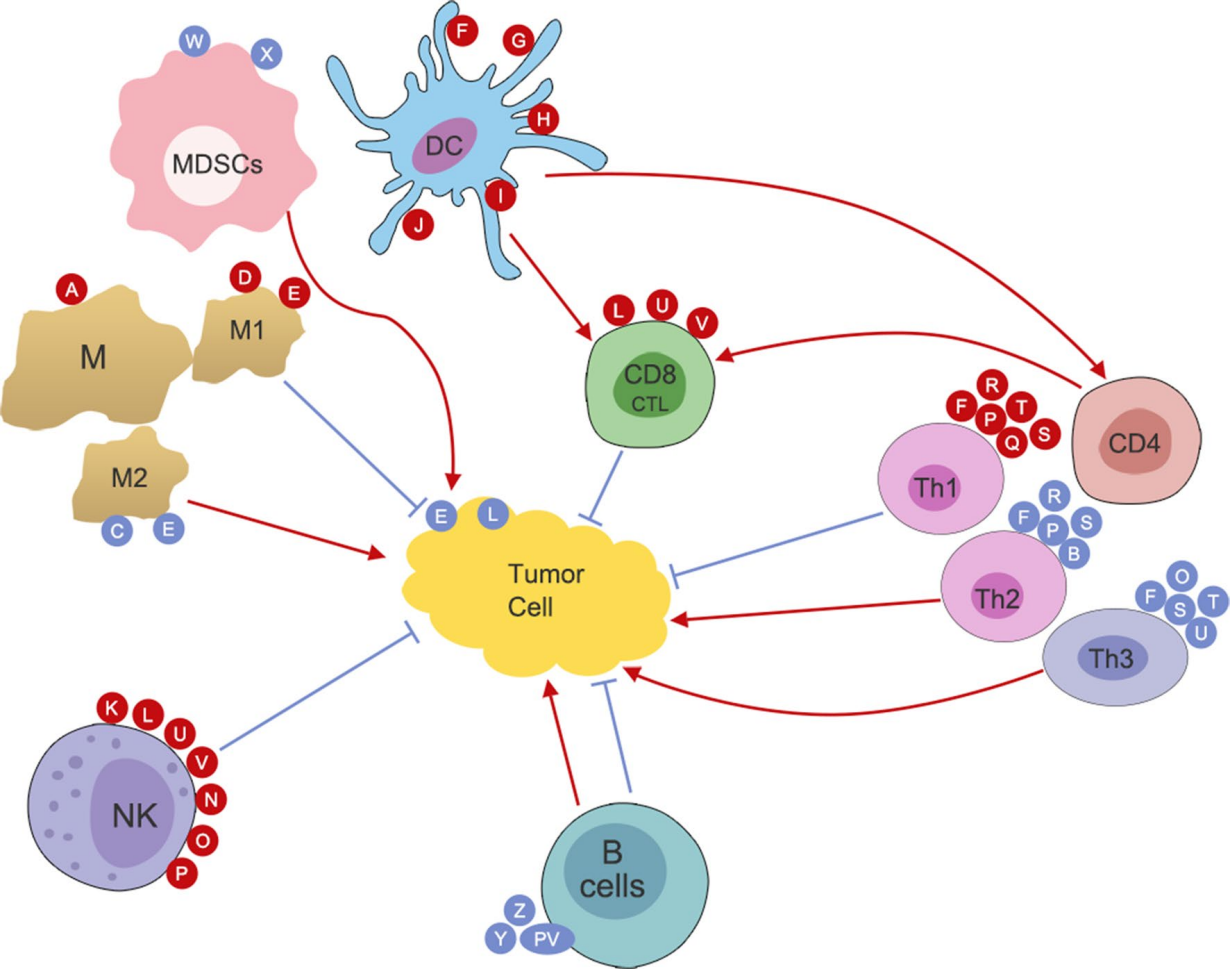
A graphic summary of anticancer immunity: innate and adaptive immune cells regulated by a variety of antitumor traditional Chinese medicine and its components. Immune cells: *M* Macrophage, *NK* Natural killer cell, *DC* Dendritic cell, *MDSC* Myeloid-derived suppressor cell. Chinese medicine and its ingredients: *A* Rhodiola, *B* Astragalus embranaceus, *C* Astragaloside IV, *D* Crassocephalum Crepidioides extract, *E* Soyasapogenols, *F* Astragalus Polysaccharides, *G* Codonopsis Polysaccharides, *H* Shikonin, *I* Achyranthes Bidentata Polysaccharides, *J* Cordyceps Sinensis, *K* lupanol, *L* ZPDC, a glycoprotein extracted from Pepper; *U* Ganoderma Polysaccharide, *V* Salviae Miltiorrhizae Polysaccharides, *N* ACNO, a Chinese herb formula, anticancer number one; *O* Echinacea, *P* Tetramethylpyrazine Phosphate, *Q* Ginsenoside, *R* HemoHIM, a herbal medicine preparation of three Chinese medicine herbs Cnidium officinale Makino, Angelica gigas Nakai, Paeonia japonica Miyabe; *S* Glycyrrhizia Polysaccharide, *T* Bushen Gubiao Recipe (BGR), *W* Icariin, *X* Asparagus Polysaccharide, *Y* Matrine, *Z* Gambogic Acid, *PV* Prunella Vulgare. The red arrows represent activation while blue lines indicate suppression, with the letters inside red or blue dots representing traditional Chinese medicine or their ingredients

In contrast to Western medicine, traditional Chinese medicine (TCM) focuses on the systematic regulation of the tumor microenvironment (TME) [17]. The main mechanism underlying the effects of TCM medications on the TME involves the adjustment of the immune system of patients with cancer [18]. The effects of TCM medications on the immune system are diverse and complex. Different TCM medications affect different immune cell types; however, some medications affect the same cell types. TCM medication types include alkaloids, polysaccharides, glucosides, and flavones—all of which have multiple biological functions with a wide range of effects on both innate and adaptive immunity (Fig. 1) [9]. TCM medications may contribute to T-cell proliferation and increase the levels of their related cytokines; they may also increase the numbers of regulatory and other T cells, thus reducing RT-related adverse reactions [19].

### RT and immunity

#### Immunomodulatory effect of RT

**Fig. 2:**
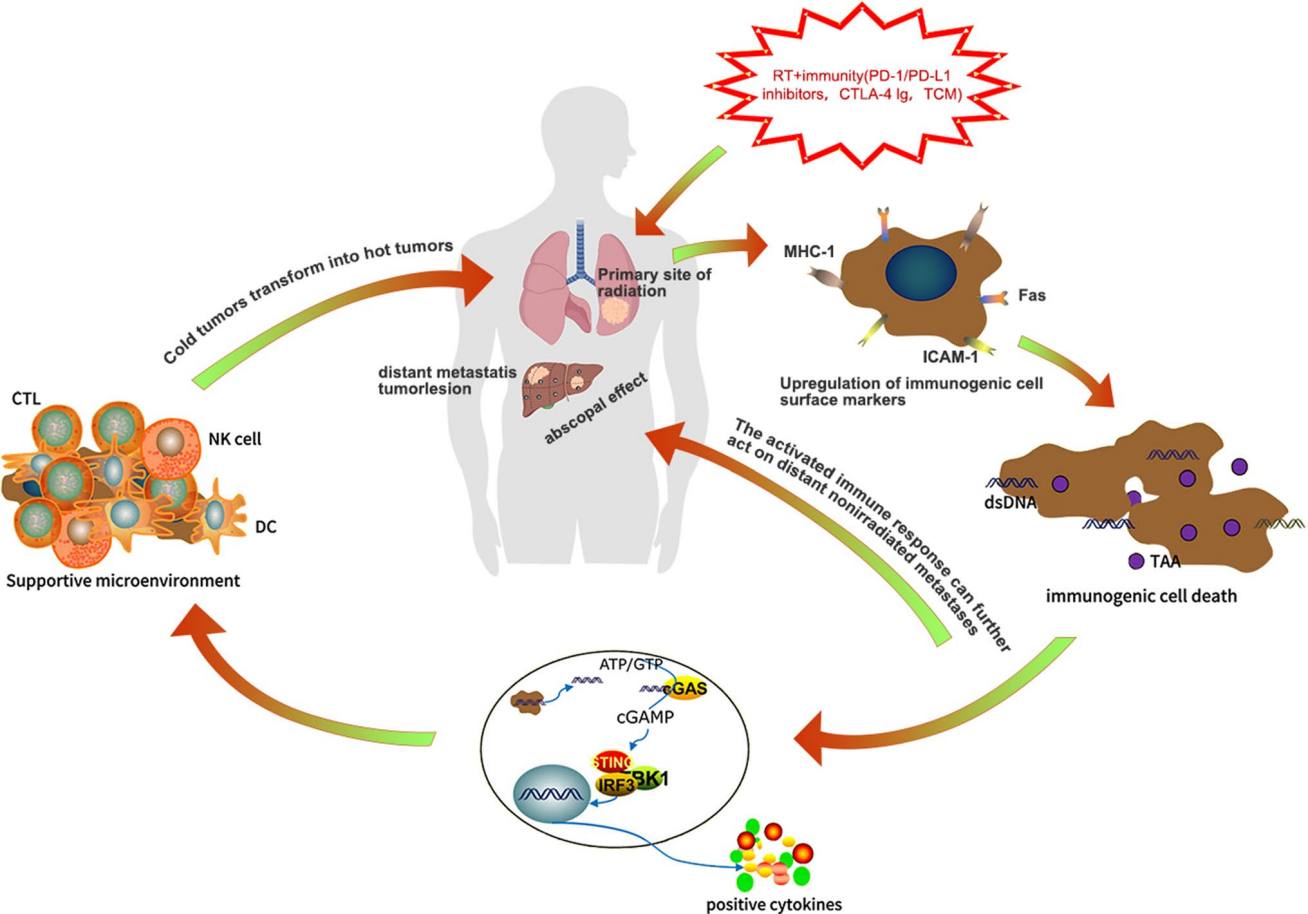
The specific mechanisms of RT combined with immunity

In addition to high cancer control efficacy, RT has demonstrated a distinct immunomodulation function in preclinical and clinical trials. RT participates in various immune regulatory processes and plays an important role in antitumor immunity (Fig. 2). In mice, tumor irradiation can elevate immunogenic cell surface markers, induce intracellular stress, especially reactive oxygen species-mediated DNA damage, leading to the occurrence of immunogenic cell death. Irradiation also results in the release of cytoplasmic DNA and stimulation of the interferon pathway, which eventually creates a proinflammatory cytokine environment [3]. The major manifestation of radiation-induced systemic immune activation is the abscopal effect, where cells that did not receive radiation also become damaged [20]. Derived from the Latin words *ab* (away from) and *scopus* (target), the abscopal effect was first described in 1953; it is defined as the regression or disappearance of a tumor outside of the radiation field but within the same organism [21]. However, the mechanisms underlying RT-induced abscopal effects remain unclear. Moreover, only 46 cases of the abscopal effect were reported between 1969 and 2014 [22]. Nevertheless, a study confirmed that the abscopal effect is unrelated to the immune system, because immunodeficient mice treated with immunotherapy did not show evidence of the effect [23].

RT may induce a systemic, immune-mediated antitumor effect by participating in the cancer-immune cycle. In general, RT results in a more favorable immunological microenvironment for antitumor immunity, transforming a cool tumor into a hot one [24].

#### RT-activated immune-related pathways

RT can induce a variety of immune-related pathways through tumor cells destruction, including the cyclic guanosine monophosphate-adenosine monophosphate synthase (cGAS)–stimulator of interferon genes (STING) pathway [25]. The cGAS-STING pathway activation, followed by interferon (IFN) α activation, induces the cross-priming ability of RT-induced dendritic cells [26]. The cGAS–STING receptors are necessary to accumulate cytoplasmic DNA, particularly in the form of micronuclei [27]. Irradiation damages tumor cells and engenders the release of nuclear DNA into the cytoplasm. The presence of mutant DNA in the cytoplasm leads to the production of the cell-cycle protein GMP-AMP, which is a cGAS product that upregulates the *IFNA* transcription through the STING-nuclear factor kappa B signal transduction [28]. In addition, RT-triggered mitochondrial outer membrane permeabilization allows for mitochondrial DNA to be exposed to the cytoplasm; it can also trigger cGAS-driven IFN-α synthesis [29]. The STING-cGAS pathway is also crucial for dendritic cells to sense irradiated tumor cells and induce adaptive immunity [30].

#### RT-related immune sensitization

Immunotherapy can regulate the TME by normalizing the vascular structure of a tumor, thereby enhancing its sensitivity to RT. Cancer immunotherapy induces the normalization of tumor vessels in a T-cell-dependent manner. In a study, when CTLA-4 Ig or PD-1 inhibitor was administered to mouse breast and colon tumor models, the sensitive tumor models demonstrated considerable growth inhibition, increased blood vessel perfusion, and reduced intratumoral hypoxia. Moreover, immunotherapy can increase vascular perfusion by promoting CD8^+^ T-cell aggregation and IFN-γ secretion, thus normalizing the tumor vascular system. The degree of tumor blood vessel normalization is closely related to its therapeutic effect [31]. A study determined the correlation between the immune stimulation pathway and vascular normalization-related genes. The results demonstrated that type 1 CD4^+^ T cells participated in the normalization of the blood vessel system, thus promoting the secretion of IFN-γ by CD4^+^ T cells (through immune checkpoint blockade), as well as supporting vascular normalization, but reducing hypoxia [32]. Therefore, tumor blood vessel normalization by ICIs can provide a feedback circuit for the immune microenvironment reprogramming, as well as enhance immunotherapy efficacy, possibly increasing the tumors’ sensitivity to RT.

Different immune treatments may have a differential effect on primary and abscopal tumor response [33]. Vaccination, for instance, is a new area of study that has produced promising outcomes for the future of immunotherapy. It can enhance RT-induced immune response and achieve immunosuppression by inhibiting immunosuppressive molecules, such as PD-1, and activating whole-tumor cell vaccines [34].

#### Synergistic activity of RT and ICIs

Many preclinical studies have shown that RT combined with ICIs may aid in systematically eradicating some diseases in a mouse model [35, 36]. Deng et al. [26] demonstrated that radiation enhanced PD-L1 expression in the TME, and radiation combined with PD-1 inhibitors efficiently suppressed cancer growth and delayed the growth of distant tumors through cytotoxic T cell activation and reduced myeloid-derived inhibitory cell accumulation in mice. Radiation combined with PD-1 inhibitors led to synergistic enhancement of their antitumor activity through CD8^+^ T cell infiltration in a mouse NSCLC model [37]. In a retrospective study, chemo-RT (CRT) led to an increase in tumor PD-L1 expression following patients with NSCLC; this result provided the pathological basis for post-CRT ICI administration [38]. Thus, the possible mechanism underlying the effects of RT with ICIs is as follows: irradiation activates the immune system against cancer cells, and then, ICIs may counteract the TME’s immunosuppression checkpoint blockade [39]. Thus far, many preclinical and clinical studies have explored the theoretical basis of iRT and maximization of its therapeutic effects. As such, iRT has been noted to demonstrate a synergistic effect of immunotherapy and RT on cancer treatment [40].

In the subsequent two sections, we discuss the efficacy and safety of different types of iRT and how they may be improved.

## iRT in NSCLC

### iRT efficacy in NSCLC

#### Efficacy of RT combined with ICIs

Recent preclinical and clinical studies have indicated that RT combined with PD-1/PD-L1 inhibitors may enhance the immune function and recover the CD8^+^ T-cell activity, thereby inhibiting tumor growth and increasing patient survival considerably (Table 1) [41]. In a randomized, multicenter phase-II trial, 92 patients with advanced NSCLC were included in either the experimental group (RT + PD-1 inhibitor) or the control group (PD-1 only). Although the differences in efficiency and survival benefits between the two groups were nonsignificant, the experimental group, particularly those with PD-L1^−^ NSCLC, demonstrated a significant increase in progression-free survival (PFS) and overall survival (OS) [42].

**Table 1:** The clinical trials exploring the efficacy of RT combined with PD1/PD-L1 in the treatment of NSCLC

NCT number	Patients	Tumor stage	PD-1/PD-L1 inhibitor	Radiotherapy planning	Treatment schedule	Outcomes		
						ORR	PFS (months)	OS (months)
NCT02492568	92	Advanced	Pembrolizumab 200 mg/kg q3w for up to 24 months	3 doses of 8 Gy	Pembrolizumab either after SBRT vs Pembrolizumab alone	18% VS 36%	mPFS 1.9 vs 6.6; *p* = 0.19	mOS 7.6 vs 15.9 *p* = 0.16
NCT02125461	713	Stage III unresectable	Durvalumab 10 mg/kg q2w	Concurrent chemoradiotherapy(60–66 Gy in 30–33 fractions)	Pembrolizumab vs placebo + chemoradiotherapy	NR	4-year PFS 35.3% vs 19.5%	4-year OS 49.6% vs 36.3%
NCT02444741	100	Metastatic	Pembrolizumab 200 mg q3w	SBRT 50 Gy in 4 fractions or RT 45 Gy in 15 fractions	Pembrolizumab + RT vs pembrolizumab alone	38%; 10% vs 33%;17%	mPFS 9.1 vs 5.1	4-year OS 49.6% vs 36.3%
NCT01295827	97	Stage IV advanced	Pembrolizumab 10 mg/kg q3w or 10 mg/kg q2w	Previously received any radiotherapy	Pembrolizumab with a history of radiotherapy vs pembrolizumab alone	NR	mPFS 4.4 vs 2.1; *p* = 0.019	mOS 10.7 vs 5.3; *p* = 0.026
NCT03285321	93	Stage III unresectable	Pembrolizumab 200 mg q3w for up to 12 months	Concurrent chemoradiotherapy (59.4–66.6 Gy)	Concurrent chemoradiation with consolidation pembrolizumab	NR	mPFS 18.7	mOS 35.8

Geng et al. [43] demonstrated that the effects of PD-1/PD-L1 inhibitor use after RT were superior to those of concomitant PD-1/PD-L1 inhibitor and RT use. These findings corroborate those of preclinical studies, suggesting that RT upregulates PD-L1 expression via DNA double-strand breakage and CD8^+^ T-cell penetration [44]. High PD-L1 levels have been demonstrated to enhance PD-1/PD-L1 activity and CD8^+^ T-cell penetration. In the phase-III PACIFIC study, durvalumab was employed as maintenance therapy after CRT in patients with unresectable stage-III pulmonary cancer, and its results demonstrated significant survival outcomes. Durvalumab treatment initiation during the first 14 days after RT termination led to longer patient survival than did durvalumab treatment initiation 14–42 days after RT termination [45].

Geng et al. [43] also demonstrated that in patients with advanced NSCLC, stereotactic-body RT (SBRT) combined with PD-1/PD-L1 inhibitors demonstrated superior outcomes to those of conventional RT combined with PD-1/PD-L1. The long-term OS related to SBRT was noted in a phase-II randomized trial only in the PD-L1^−^ NSCLC population [42], suggesting that RT, particularly SBRT, modifies PD-L1 expression and thus enhances ICI effectiveness. In addition to activating the immune system, RT can induce lymphopenia, which may also enhance the effectiveness of ICIs [43]. The major factors underlying the development of RT-related lymphopenia include the use of multicenter or multisite radiation, high RT doses, as well as differences in RT technologies used; SBRT is prone to reduce this risk [46]. The beneficial effects of combined therapy with SBRT and PD-1/PD-L1 inhibitors are attributable to the aforementioned benefits of SBRT. SBRT and particle-beam RT can aid in reducing exposure to normal tissues and activating the immune system, making it the preferred choice for iRT [47].

In general, iRT can enhance OS, PFS, and tumor response in patients with advanced NSCLC. PD-1/PD-L1 inhibitor administration following RT or precise radiation treatments, such as SBRT, are likely to provide more benefits for those treated with combined treatments. These findings require additional large-scale, randomized, controlled trials to verify.

#### Efficacy of RT combined with TCM medications

Through health qi strengthening and pathogen removal, TCM improves RT efficacy, eventually promoting carcinoma cell apoptosis, suppressing tumor metastasis, strengthening antitumor immune function, adjusting TME stability, improving RT efficacy, and reducing recurrence rate [48, 49]. Kong et al. [50] discovered that micheliolide increases radiosensitivity by inducing the ubiquitination degradation of hypoxia-inducible factor-1α in p53-positive NSCLC. Yu et al. [51] reported that ganoderma lucidum polysaccharide combined with RT reduces tumor immunosuppression. Xiao et al. [52] showed that Aidi injection and RT could significantly improve the clinical efficacy and quality of life of patients with lung cancer. However, additional clinical studies are warranted to increase the current knowledge regarding iRT and improve the survival outcomes of patients with NSCLC.

### iRT safety

#### Safety of RT combined with ICIs

Because RT can induce local and systemic inflammatory responses, iRT may be associated with increased toxicity. Several retrospective and prospective single-arm studies on the safety of RT combined with ICIs have been reported so far [53]. Marco et al. [54] pooled clinical data of 187 NSCLC patients treated with concurrent RT and PD-1/PD-L1 inhibitors at seven Italian centers between September 2015 and June 2019; the patients’ median follow-up duration was 23 months, whereas their median OS was 16.5 months. Of all 187 patients, 13 and 43 demonstrated RT- and immune-related adverse reactions, respectively. In most cases, RT and PD-1/PD-L1 inhibitors did not lead to toxic effects additively; however, one patient demonstrated grade 5 pulmonary toxicity—a possible negative consequence of RT combined with the ICIs. These results indicate that, in general, the combined use of RT and PD-1/PD-L1 inhibitors is safe; this combination does not worsen their individual toxicities. Despite the potential bias, a large-scale, retrospective, multicenter, observational cohort trial on sub-directional RT combined with ICIs demonstrated that concomitant use of palliative and ablative RT schemes is safe. This finding may be relevant because the study included a large number of patients with NSCLC demonstrating satisfactory results and relatively long-term survival; additional studies on optimizing this combination are therefore warranted [54]. In their systematic review, Sha et al. [55] compared the toxic effects of ICIs alone with RT combined with ICIs by including 51 trials (phase-III or beyond), including a total of > 15,000 patients. The results indicated that 17.8% of the patients who received RT combined with ICIs demonstrated grade 3 or higher toxicity. In contrast, 22.3% of the patients who received ICIs alone experienced grade 3 or higher toxicity. In addition, the differences in toxicity concerning irradiation location (intracranial versus extracranial) or RT sequencing were nonsignificant.

RT combined with CTLA-4 Ig can lead to grade 3 or higher toxicity [56]. Formenti et al. [57] reported the first and only phase-I/II prospective trial on the use of SBRT combined with a high dose (3 mg/kg) of ipilimumab (a CTLA-4 Ig) for metastatic lung cancer detection; its results indicated that 38% of the patients demonstrated grade 3 or higher toxicity. This toxicity may have been due to the high ipilimumab dose rather than due to SBRT. Low-dose CTLA-4 Igs are effective and less toxic, and the effects of their combination with stereotactic ablative RT (SABR). In their systematic review, Sha et al. reported that CTLA-4 Ig alone led to significantly higher toxicity than PD-1/PDL-1 inhibitors [55].

A phase-II randomized clinical trial also demonstrated acceptable safety, with 30% and 20% of patients who received ICIs alone and SBRT combined with ICIs experiencing grade 3 or higher toxicity, respectively [42]. In their systematic analysis of prospective and retrospective trials, Chicas-Sett et al. [58] investigated the effects of SBRT combined with ICIs in patients with metastatic NSCLC and reported that the PD-1/PD-L1 inhibitor alone and SBRT combined with ICIs led to similar toxicity.

The safety of iRT, therefore, depends on the ICIs, along with their dosage and dosing frequency. In general, iRT and ICIs alone appear to have similar toxicity. However, the available information is only provisional, with most data not being from prospective trials. However, evidence on the effects of treatment variables on toxicity characteristics is lacking, and further relevant research to validate these results is required.

#### Safety of RT combined with TCM medications

TCM medications suppress tumor growth and enhance immunity. Accumulating evidence suggests that a variety of herbal medicines play an important role in reducing toxicity and increasing efficacy of radiotherapy for NSCLC (Table 2). From the perspective of immune regulation, RT combined with TCM medications may have clinical utility. Wu et al. [59] conducted a controlled clinical trial on the efficacy and safety of CRT combined with Zengxiao Jiandu decoction in the treatment of unresectable, locally advanced NSCLC. From February 2019 to December 2020, the authors randomly included 163 cases in the TCM or control group. In the TCM group, 59 patients completed CRT according to the protocol, whereas 79 received Zengxiao Jiandu decoction according to the protocol. In the control group, 42 individuals completed the CRT according to the schedule, and their rates of grade 3 or higher CRT-related toxicity were greater than those in the TCM group (44.4% vs. 31.7%). Grade III radiopneumonitis incidence was higher in the control group than in the TCM group (13.6% vs. 3.7%); similarly, the mean PFS was longer in the TCM group than in the control group (12.0 vs. 9.0 months). In general, Zengxiao Jiandu decoction as an add-on treatment reduced treatment-related toxicity, improved CRT completion rate, and extended PFS in patients with unresectable locally advanced NSCLC. These results supported the widespread application of Zengxiao Jiandu soup in the treatment of unresectable locally advanced NSCLC [59]. Zhang et al. found that Guiqi Baizhu Decoction composed of Radix Astragali seu Hedysari, Radix Angelicae Sinensis, Rhizoma Atractylodis, Radix Paeoniae Alba, Pericarpium Citri Reticulatae, Radix et Rhizoma Rhei, and Radix Glycyrrhizae can reduce radiation-induced inflammatory response and immune injury and prevent intestinal microbial imbalance and metabolic disorders caused by RT significantly. [60]. Accumulating clinical evidence indicates that RT combined with TCM extracts or formulas related to the immune system can improve treatment safety significantly.

**Table 2:** The safety of natural compound/traditional Chinese medicine combined with RT in NSCLC

Natural compound/Traditional Chinese medicine	Type of drug	Experimental models/(preclinical or clinical)	Function
Zengxiao Jiandu decoction	Compound prescription, composed of grams Radix glehniae, grams Radix Ophiopogonis, grams Astragalus root, grams Radix Notoginseng, grams Caulis Spatholobi, grams Pyrrosia lingua, grams Angelica sinensis, grams Fructus lycii and grams Rhizoma polygonate	Clinical	Significantly reduced the occurrence of grade ≥ 3 radiation pneumonitis
Ren-Shen	Monomer herb	Clinical	Ameliorating cancer-related fatigue without any discernible toxicity
Radix Codonopsis	Monomer herb	Clinical	Reducing the immunosuppressive effect of radiotherapy, reduce myelosuppression
Curcumin	Herbal extractions from Curcumin	Preclinical	Protects normal organs such as liver, kidney, oral mucosa, and heart from chemotherapy and radiotherapy-induced toxicity
Bu-Zhong-Yi-Qi Decoction	Compound prescription, composed of pinellia tuber, Scutellaria baicalensis, Zingiberis rhizoma, Zizyphi fructus, Coptidis rhizoma, Glycyrrhiza radix, Panax ginseng	Clinical	(1) Have protective effect of intestine and hematopoietic organs against radiation damage. (2) Improving localized radiotherapy-induced immune deterioration. (3) Improving cancer-related fatigue and QOL. (4) Reducing radiation or chemotherapy induced side effects
Shenqi fuzheng injection	Compound prescription, composed of extracts from radix Astragali Codonopsis pilosula	Preclinical	Attenuate cranial radiation therapy-induced brain injury in mice via inhibition of the NF-κB signaling pathway and microglial activation

Many clinical trials have suggested that iRT for NSCLC does not increase toxicity significantly, with acceptable safety. In general, more robust, longer-term, randomized, prospective studies with stricter follow-ups that may aid in gaining an overall picture of the undesirable effects of combined therapy are warranted.

### iRT limitations

#### Optimal radiation dose and fractionation

Despite the increasingly widespread use of RT, consensus has not been reached regarding the recommended RT dosages and segmentation methods. Both preclinical and clinical trials have investigated many treatment methods for their anticancer efficacy; however, no conclusions have been reached [41].

RT segmentation schemes, conventionally, can be either conventional (1.8–2.2 Gy/time, once/day, 5 days/week, 3–7 weeks in total) or large (including stereotactic radiosurgery; 3–20 Gy/time, one fraction/day) [61]. Siva et al. [62] indicated that conventional fractionation schemes of RT might facilitate radiation-induced antitumor immunity. The authors also found that a single high-dose (12 Gy) RT does not exhaust established immune effector cells, such as CD8^+^ T cells or natural killer (NK) cells, and that it might be more effective in killing cancer cells when combined with immunotherapy. SBRT, a typical type of hypo-RT, consists of high-dose narrow edge and strong gradient RT (i.e., SABR) that protect adjacent healthy tissues [40]. Hypofractionation, particularly with stereotactic RT, may facilitate the reduction of exposure to the nonintervenient parts of the heart and lungs to maintain absolute lymphocyte count effectively and elicit a relatively robust abscopal response [63]. Although each large dose may increase abscopal responses, clinical studies have not achieved good results, suggesting that the abscopal effect is affected by several factors [61].

Herrera et al. [64] indicated that when used to irradiate all detectable tumors, low-dose irradiation (LDI) with a wide external-beam irradiation field can efficiently mobilize innate and adaptive immunity in the presence of an immune suppression pathway; LDI is, therefore, suitable for combining with an ICI. A post hoc analysis of three iRT studies with a CTLA-4 Ig or a monoclonal antibody against PD-1/PD-L1 was reported recently. According to its evaluation criteria for solid tumor response, 58% of patients who received LDI scattering from high-dose RT fields demonstrated a partial or complete response. In contrast, only 18% of patients who did not receive scattered LDI demonstrated a partial or complete response [65]. Olza et al. demonstrated that LDI provides good security; it can irradiate several or even all tumor deposits. Similar to SBRT, LDI may be used in combination with immunotherapies. These combinations may enhance T-cell initiation and activation and decrease TME immunity suppression. Finally, combined treatment with high-dose iRT for several metastatic lesions to trigger in situ vaccination, followed by LDI for residual metastatic lesions, may maximize the abscopal effects of the treatment through the facilitation of T-cell invasion [66]. Although LDI does not kill cancer cells, it can be used to enhance immunotherapy efficacy through the activation of immunity and TME regulation. A recent study on SBRT combined with ipilimumab for advanced metastatic cancer treatment demonstrated that compared with lesions far from the target tumor, tumors exposed to LDI (due to proximity to the target tumor) were more likely to react [67]. On the basis of these results, Shang et al. proposed a novel therapeutic approach, integrating high-dose RT with LDI to enhance the therapeutic effect of whole-body immunotherapy [33]. High-dose RT can enhance antigen expression and release and stimulate immune cell activation, whereas LDI facilitates immune cell penetration into the distant stroma and tumor bed. In a phase-II nonrandomized study, patients who received high-dose iRT combined with LDI demonstrated more local reactions than those who did not receive LDI [68].

As part of an iRT paradigm, LDI is a novel approach addressing the mechanical constraints of high-dose RT; however, further confirmation of the aforementioned results is warranted.

### iRT sequence

The optimal drug administration plans for RT combined with ICIs are currently unclear. In particular, whether ICIs be administered concurrently or sequentially with RT requires further investigation. This time window might significantly impact the antitumor effect of the combined therapy [61]. Some literature suggests that sequencing may rely on the ICI type [69].

Clinical studies have shown that PD-L1 inhibitor therapy may be more effective when used simultaneously with or after RT. Recently, Antonia et al. [70] demonstrated the survival advantages of using duvacumab after synchronous CRT in patients with grade III, unresectable NSCLC. In a phase-2 randomized clinical trial, Willemijn et al. indicated that SBRT before pembrolizumab is well tolerated [42]. In their retrospective study, Susan et al. [71] demonstrated that the OS rate may be lower in patients who have completed immunotherapy before SBRT than in those treated simultaneously or subsequently. The authors also reported a longer median OS in patients treated with immunotherapy and SBRT simultaneously than in those treated with immunotherapy 6 months before SBRT; however, this difference was only partially explained because of potential lead-time bias. Therefore, additional prospective cohort studies with causal analysis of death and toxicity are required to validate this analysis. Bauml et al. [72] demonstrated that pembrolizumab after local ablative treatment resulted in a significant increase in PFS relative to historical data in patients with oligometastatic NSCLC without any decline in quality of life. A phase-III, placebo-controlled study demonstrated that durvalumab after CRT in patients with unresectable stage-III NSCLC led to sustained PFS and OS benefits. Of all patients, 49.6% had an OS of ≥ 4 years, and 35.3% demonstrated no progression [45]. In their nonrandomized experimental study, Salma et al. reported that concomitant pembrolizumab and CRT have strong antitumor activity with tolerable toxicity, regardless of tumor histology and PD-L1 tumor fraction score [73].

Many animal studies on the ideal sequence for administering CTLA-4 Igs and RT have indicated that CTLA-4 Ig treatment has superior anticancer effects when administered before RT [74, 75]. In their preclinical trial, Young et al. [75] administered CTLA-4 Igs 7 days before RT (20 Gy × 1), 1 day after RT (20 Gy × 1), or 5 days after RT (20 Gy × 1) and noted that administration of CTLA-4 Ig before RT led to optimal outcomes. The authors also investigated the use of antiOx40 (a secondary co-stimulus checkpoint inhibitor) with identical RT sequences and observed that ICIs were most beneficial when they were administered the first day after SBRT. The authors conclude that the effect of sequencing depends on the mechanism of immunotherapy being used. These differences may be due to PD-1/PD-L1 inhibitors reduce the number of newly activated T cells [76], whereas CTLA-4 Ig acts on naïve and regulatory T cells [77]. In general, the optimal iRT sequence may depend on the immunomodulators’ types.

Despite scant data, the available results demonstrate that PD-1/PD-L1 inhibitors may be more effective when administered simultaneously or after RT, whereas CTLA-4 Igs may be more effective when administered before RT. However, whether these immunomodulators achieve their highest efficacy by simultaneously inducing increased toxicity levels warrants additional prospective head-to-head clinical trials with comprehensive procedures.

### Patient selection and biomarkers

iRT may not be equally beneficial to all patients with cancer [33]. Currently, reliable biomarkers or models for predicting immune therapy or combination therapy responses remain unavailable [39]. Because of the breakthrough progress of PD-1/PD-L1 inhibitor use in cancer treatment, studies on their prognostic markers are ongoing. Thus far, many biomarkers that predict survival outcomes have been reported; however, no definitive standard or effectiveness has been identified [41].

PD-L1 positivity is currently the most accepted marker of immunotherapy response [78, 79]. Reck et al. [80] demonstrated that pembrolizumab is associated with increased PFS and OS as well as a reduction in the incidence of treatment-related adverse events in advanced NSCLC patients who have not received prior treatment or have a PD-L1^+^ tumor rate of > 50%. In their post hoc exploratory analysis, Bang et al. [81] reported that compared with the placebo, durvalumab affords significant improvements in PFS and OS in patients with a PD-L1^+^ tumor rate of > 1%; however, such considerable improvement was not observed in patients with a PD-L1^+^ tumor rate of < 1%.

Tumor mutation burden (TMB) is another major biomarker for immune response, and this marker has been correlated with the response rate of PD-1 or PD-L1 inhibitor therapy in various cancer types [82, 83]. However, TMB has been applied and validated less extensively than PD-L1 positivity [33]. In 2018, TMB was written into the first edition of the National Comprehensive Cancer Network’s (NCCN’s) NSCLC guidelines, where it was identified as an emerging biomarker for patients with metastatic NSCLC. However, on the basis of subsequent clinical experimental data, TMB may be associated with limitations, such as poor detection repeatability and difficulty when the TMB is high. Therefore, in 2020, NCCN expert group unlisted TMB as an emerging biomarker for metastatic NSCLC. In the current (i.e., fifth) edition of the NCCN guidelines, TMB detection is recommended as a biomarker for ICI selection only in advanced NSCLC patients who have not received immunotherapy previously. TMB is considered an efficacy marker for second-line and post-second-line immunotherapy but not first-line immunotherapy. In summary, TMB is a biomarker with potential clinical application value; however, it has several limitations, and its specific application value remains unclear [84].

Mismatch repair deficiency and microsatellite instability may accurately predict the efficacy of PD-1/PD-L1 inhibitors [85, 86]. Moreover, tumor-infiltrating lymphocytes, particularly CD8^+^ T cells, and cytokines are potential biomarkers for inhibitor therapy [87, 88]. Radiomics is a powerful, noninvasive, economical, and reliable methodology for evaluating patient response to precision medicine [89]. Radiomics strategies provide longitudinal monitoring of tumor characteristics; they can be combined with tumor biopsy and genome sequencing to improve treatment selection. Systemic inflammatory indicators, including the proportion of neutrophils to platelets, lymphocyte ratio, and NK cell count, may be reliable predictors of SBRT outcomes [90].

Finally, the immune function of specific patients may be related to iRT benefits. For iRT to exert its downstream effects, adequate immune function, particularly lymphocyte function, is essential. Data from small-scale, less-reliable data sets have demonstrated that patients with insufficient immune function (such as those with lymphocytopenia) are less likely to benefit from iRT [63, 91].

In general, selecting suitable patients can improve iRT effectiveness. Moreover, several clinical and pathological factors facilitate patient selection for iRT, thereby improving treatment efficacy and NSCLC prognosis.

## Future directions

According to most preclinical and clinical trials reported thus far, RT combined with ICIs is a promising option for NSCLC treatment. Currently, many clinical studies have explored various therapeutic approaches, such as using different dosages and fractionation schemes, along with biomarkers and techniques for patient selection. Future clinical trials providing conclusive evidence for the beneficial effects of combined therapy in NSCLC are required. RT is a better choice for personalized cancer vaccination, which can be applied to a wide range of populations and tumor types. Moreover, studies on different RT modes, such as SBRT, high-linear-energy transfer RT, charged particle therapy, and high-dose-rate brachytherapy, are underway [92].

Clinical trials exploring newer RT treatments have also been reported; with the emergence of newer RT modes, studies have compared the immune regulation outcomes of FLASH RT with heavy ion beams with those of photon-beam RT in [53]. According to Brooks et al. [93], the maximum abscopal effect may be achieved by irradiating more than one lesion because this technique may address tumor heterogeneity, metastatic lesion clonality, and differences in lesion immunogenicity or local immunosuppressive effects, as well as reduce tumor burden. Multisite RT combined with ICIs can enhance anticancer efficacy in patients with metastatic NSCLC [94]; however, relevant clinical research is required. RT application and research have broad prospects: evidence over 2012–2022 has indicated that RT and immunotherapy simultaneously act on solid tumors, including NSCLC. Nevertheless, many relevant studies are ongoing; therefore, additional evidence supporting iRT use in NSCLC may be reported over the next few years.

## Conclusion

In this review, we listed and discussed studies on the efficacy and safety of several iRT strategies for NSCLC treatment. The results indicated that immunotherapy after RT leads to optimal efficacy in patients with NSCLC. Moreover, its safety depends on the ICI type, dose, and frequency. Furthermore, LDI, an emerging method, can resolve the mechanism-related limitations of high-dose RT, as part of the iRT paradigm. Finally, the optimal dosing schedule for RT in combination with ICIs may depend on the type of ICI. In summary, strategies for improving iRT efficacy and safety in a wider range of patients with NSCLC warrant development.

## Data Availability

Data sharing is not applicable to this article as no new data were created or analyzed in this study.

## References

[1] Siegel RL, Miller KD, Wagle NS, Jemal A (2023). Cancer statistics, 2023. CA Cancer J Clin.

[2] Garon EB, Hellmann MD, Rizvi NA, Carcereny E, Leighl NB, Ahn MJ (2019). Five-year overall survival for patients with advanced non-small-cell lung cancer treatepd with pembrolizumab: results from the phase I KEYNOTE-001 study. J Clin Oncol.

[3] Fitzgerald K, Simone CB (2020). Combining immunotherapy with radiation therapy in non-small cell lung cancer. Thorac Surg Clin.

[4] Lin AJ, Roach M, Bradley J, Robinson C (2019). Combining stereotactic body radiation therapy with immunotherapy: current data and future directions. Transl Lung Cancer Res.

[5] Siegel RL, Miller KD, Wagle NS Jemal A. Cancer statistics, 2023. CA Cancer J Clin. 2023; 73(1): 17–48. 10.3322/caac.21763 36633525

[6] Bradley JD, Hu C, Komaki RR, Masters GA, Blumenschein GR, Schild SE (2020). Long-term results of NRG oncology RTOG 0617: Standard- versus high-dose chemoradiotherapy with or without cetuximab for unresectable stage III non-small-cell lung cancer. J Clin Oncol.

[7] Wirsdorfer F, de Leve S, Jendrossek V (2018). Combining radiotherapy and immunotherapy in lung cancer: can we expect limitations due to altered normal tissue toxicity?. Int J Mol Sci.

[8] Roh M, Wainwright DA, Wu JD, Wan Y, Zhang B (2020). Targeting CD73 to augment cancer immunotherapy. Curr Opin Pharmacol.

[9] Wang Y, Zhang Q, Chen Y, Liang CL, Liu H, Qiu F (2020). Antitumor effects of immunity-enhancing traditional Chinese medicine. Biomed Pharmacother.

[10] Hellmann MD, Paz-Ares L, Bernabe Caro R, Zurawski B, Kim SW, Carcereny Costa E (2019). Nivolumab plus ipilimumab in advanced non-small-cell lung cancer. N Engl J Med.

[11] Reck M, Rodríguez-Abreu D, Robinson AG, Hui R, Csőszi T, Fülöp A (2021). Five-year outcomes with pembrolizumab versus chemotherapy for metastatic non-small-cell lung cancer with PD-L1 tumor proportion score ≥ 50. J Clin Oncol.

[12] Jassem J, de Marinis F, Giaccone G, Vergnenegre A, Barrios CH, Morise M (2021). Updated overall survival analysis from IMpower110: Atezolizumab versus platinum-based chemotherapy in treatment-naive pgrammed death-ligand 1-selected NSCLC. J Thorac Oncol.

[13] Herbst RS, Giaccone G, de Marinis F, Reinmuth N, Vergnenegre A, Barrios CH (2020). Atezolizumab for first-line treatment of PD-L1-selected patients with NSCLC. N Engl J Med.

[14] Reck M, Rodríguez-Abreu D, Robinson A, Hui R, Csőszi T, Fülöp A (2016). Pembrolizumab versus chemotherapy for PD-L1-Positive non-small-cell lung cancer. N Engl J Med.

[15] O'Donnell JS, Teng MWL, Smyth MJ (2019). Cancer immunoediting and resistance to T cell-based immunotherapy. Nat Rev Clin Oncol.

[16] Chicas-Sett R, Zafra-Martin J, Morales-Orue I, Castilla-Martinez J, Berenguer-Frances MA, Gonzalez-Rodriguez E (2020). Immunoradiotherapy as an effective therapeutic strategy in lung cancer: From palliative care to curative intent. Cancers (Basel).

[17] Zhang Y, Lou Y, Wang J, Yu C, Shen W (2020). Research status and molecular mechanism of the traditional Chinese medicine and antitumor therapy combined strategy based on tumor microenvironment. Front Immunol.

[18] Wang S, Long S, Deng Z, Wu W (2020). Positive role of chinese herbal medicine in cancer immune regulation. Am J Chin Med.

[19] Hoffman RD, Li CY, He K, Wu X, He BC, He TC (2020). Chinese herbal medicine and its regulatory effects on tumor related T cells. Front Pharmacol.

[20] Heeran AB, Berrigan HP, O'Sullivan J (2019). The radiation-induced bystander effect (RIBE) and its connections with the hallmarks of cancer. Radiat Res.

[21] Mole RH (1953). Whole body irradiation; radiobiology or medicine?. Br J Radiol.

[22] Abuodeh Y, Venkat P, Kim S (2016). Systematic review of case reports on the abscopal effect. Curr Probl Cancer.

[23] Demaria S, Ng B, Devitt ML, Babb JS, Kawashima N, Liebes L (2004). Ionizing radiation inhibition of distant untreated tumors (abscopal effect) is immune mediated. Int J Radiat Oncol Biol Phys.

[24] McLaughlin M, Patin EC, Pedersen M, Wilkins A, Dillon MT, Melcher AA (2020). Inflammatory microenvironment remodelling by tumour cells after radiotherapy. Nat Rev Cancer.

[25] Kwon J, Bakhoum SF (2020). The cytosolic DNA-sensing cGAS-STING pathway in cancer. Cancer Discov.

[26] Deng L, Liang H, Burnette B, Beckett M, Darga T, Weichselbaum RR (2014). Irradiation and anti-PD-L1 treatment synergistically promote antitumor immunity in mice. J Clin Invest.

[27] Ablasser A, Chen ZJ. cGAS in action: expanding roles in immunity and inflammation. Science. 2019;363(6431):eaat8657. 10.1126/science.aat8657 30846571

[28] Galluzzi L, Vanpouille-Box C, Bakhoum SF, Demaria S (2018). SnapShot: CGAS-STING signaling. Cell.

[29] Yamazaki T, Galluzzi L (2020). Mitochondrial control of innate immune signaling by irradiated cancer cells. Oncoimmunology.

[30] Li A, Yi M, Qin S, Song Y, Chu Q, Wu K (2019). Activating cGAS-STING pathway for the optimal effect of cancer immunotherapy. J Hematol Oncol.

[31] Zheng X, Fang Z, Liu X, Deng S, Zhou P, Wang X (2018). Increased vessel perfusion predicts the efficacy of immune checkpoint blockade. J Clin Invest.

[32] Tian L, Goldstein A, Wang H, Ching Lo H, Sun Kim I, Welte T (2017). Mutual regulation of tumour vessel normalization and immunostimulatory reprogramming. Nature.

[33] Shang S, Liu J, Verma V, Wu M, Welsh J, Yu J (2021). Combined treatment of non-small cell lung cancer using radiotherapy and immunotherapy: challenges and updates. Cancer Commun (Lond).

[34] Ruckert M, Deloch L, Frey B, Schlucker E, Fietkau R, Gaipl US. Combinations of radiotherapy with vaccination and immune checkpoint inhibition differently affect primary and abscopal tumor growth and the tumor microenvironment. Cancers (Basel). 2021;13(4):714. 10.3390/cancers13040714 PMC7916259 33572437

[35] Demaria S, Pilones KA, Formenti SC, Dustin ML (2013). Exploiting the stress response to radiation to sensitize poorly immunogenic tumors to anti-CTLA-4 treatment. Oncoimmunology.

[36] Dewan MZ, Galloway AE, Kawashima N, Dewyngaert JK, Babb JS, Formenti SC (2009). Fractionated but not single-dose radiotherapy induces an immune-mediated abscopal effect when combined with anti-CTLA-4 antibody. Clin Cancer Res.

[37] Skoulidis F, Goldberg ME, Greenawalt DM, Hellmann MD, Awad MM, Gainor JF (2018). STK11/LKB1 mutations and PD-1 inhibitor resistance in KRAS-Mutant lung adenocarcinoma. Cancer Discov.

[38] Yoneda K, Kuwata T, Kanayama M, Mori M, Kawanami T, Yatera K (2019). Alteration in tumoural PD-L1 expression and stromal CD8-positive tumour-infiltrating lymphocytes after concurrent chemo-radiotherapy for non-small cell lung cancer. Br J Cancer.

[39] Xia WY, Feng W, Zhang CC, Shen YJ, Zhang Q, Yu W (2020). Radiotherapy for non-small cell lung cancer in the immunotherapy era: the opportunity and challenge-a narrative review. Transl Lung Cancer Res.

[40] Wu L, Zhang Z, Bai M, Yan Y, Yu J, Xu Y (2023). Radiation combined with immune checkpoint inhibitors for unresectable locally advanced non-small cell lung cancer: synergistic mechanisms, current state, challenges, and orientations. Cell Commun Signal.

[41] Chen Y, Gao M, Huang Z, Yu J, Meng X (2020). SBRT combined with PD-1/PD-L1 inhibitors in NSCLC treatment: a focus on the mechanisms, advances, and future challenges. J Hematol Oncol.

[42] Theelen W, Peulen HMU, Lalezari F, van der Noort V, de Vries JF, Aerts J (2019). Effect of pembrolizumab after stereotactic body radiotherapy vs pembrolizumab alone on tumor response in patients with advanced non-small cell lung cancer: results of the PEMBRO-RT phase 2 randomized clinical trial. JAMA Oncol.

[43] Geng Y, Zhang Q, Feng S, Li C, Wang L, Zhao X (2021). Safety and efficacy of PD-1/PD-L1 inhibitors combined with radiotherapy in patients with non-small-cell lung cancer: a systematic review and meta-analysis. Cancer Med.

[44] Choe EA, Cha YJ, Kim JH, Pyo KH, Hong MH, Park SY (2019). Dynamic changes in PD-L1 expression and CD8(+) T cell infiltration in non-small cell lung cancer following chemoradiation therapy. Lung Cancer.

[45] Faivre-Finn C, Vicente D, Kurata T, Planchard D, Paz-Ares L, Vansteenkiste JF (2021). Four-year survival with durvalumab after chemoradiotherapy in stage III NSCLC-an update from the PACIFIC trial. J Thorac Oncol.

[46] Cho Y, Park S, Byun HK, Lee CG, Cho J, Hong MH (2019). Impact of treatment-related lymphopenia on immunotherapy for advanced non-small cell lung cancer. Int J Radiat Oncol Biol Phys.

[47] Vanpouille-Box C, Formenti SC, Demaria S (2018). Toward precision radiotherapy for use with immune checkpoint blockers. Clin Cancer Res.

[48] Liu W, Yang B, Yang L, Kaur J, Jessop C, Fadhil R (2019). Therapeutic effects of ten commonly used Chinese herbs and their bioactive compounds on cancers. Evid Based Complement Altern Med.

[49] Yang Y, Li N, Wang TM, Di L (2021). Natural products with activity against lung cancer: a review focusing on the tumor microenvironment. Int J Mol Sci.

[50] Kong P, Yu KN, Yang M, Almahi WA, Nie L, Chen G (2020). Micheliolide enhances radiosensitivities of p53-deficient non-small-cell lung cancer via promoting HIF-1alpha degradation. Int J Mol Sci.

[51] Yu H, Yang Y, Jiang T, Zhang X, Zhao Y, Pang G (2019). Effective radiotherapy in tumor assisted by ganoderma lucidum polysaccharide-conjugated bismuth sulfide nanoparticles through radiosensitization and dendritic cell activation. ACS Appl Mater Interfaces.

[52] Xiao Z, Wang C, Zhou R, Hu S, Yi N, Feng J (2018). Can aidi injection improve overall survival in patients with non-small cell lung cancer? A systematic review and meta-analysis of 25 randomized controlled trials. Complement Ther Med.

[53] Theelen WS, de Jong MC, Baas P (2020). Synergizing systemic responses by combining immunotherapy with radiotherapy in metastatic non-small cell lung cancer: the potential of the abscopal effect. Lung Cancer.

[54] Perna M, Scotti V, Ciammella P, Borghetti P, D'Angelo E, Levra NG (2021). The NIPRO study: an observational, retrospective, multicenter study on the safety of the radiotherapy and immunotherapy combination for advanced-stage NSCLC. Clin Lung Cancer.

[55] Sha CM, Lehrer EJ, Hwang C, Trifiletti DM, Mackley HB, Drabick JJ (2020). Toxicity in combination immune checkpoint inhibitor and radiation therapy: a systematic review and meta-analysis. Radiother Oncol.

[56] Karantanos T, Karanika S, Seth B, Gignac G (2019). The absolute lymphocyte count can predict the overall survival of patients with non-small cell lung cancer on nivolumab: a clinical study. Clin Transl Oncol.

[57] Formenti SC, Rudqvist NP, Golden E, Cooper B, Wennerberg E, Lhuillier C (2018). Radiotherapy induces responses of lung cancer to CTLA-4 blockade. Nat Med.

[58] Chicas-Sett R, Morales-Orue I, Castilla-Martinez J, Zafra-Martin J, Kannemann A, Blanco J (2019). Stereotactic ablative radiotherapy combined with immune checkpoint inhibitors reboots the immune response assisted by immunotherapy in metastatic lung cancer: a systematic review. Int J Mol Sci.

[59] Wu L, Zhu Y, Yuan X, Liu Y, Wu Q, Xu Q (2022). The efficacy and safety of Zengxiao Jiandu decoction combined with definitive concurrent chemoradiotherapy for unresectable locally advanced non-small cell lung cancer: a randomized, double-blind, placebo-controlled clinical trial. Ann Transl Med.

[60] Zhang LY, Zhou T, Zhang YM, Xu XM, Li YY, Wei KX (2020). Guiqi baizhu decoction alleviates radiation inflammation in rats by modulating the composition of the gut microbiota. Evid Based Complement Altern Med.

[61] Liu Y, Dong Y, Kong L, Shi F, Zhu H, Yu J (2018). Abscopal effect of radiotherapy combined with immune checkpoint inhibitors. J Hematol Oncol.

[62] Siva S, MacManus MP, Martin RF, Martin OA (2015). Abscopal effects of radiation therapy: a clinical review for the radiobiologist. Cancer Lett.

[63] Chen D, Verma V, Patel RR, Barsoumian HB, Cortez MA, Welsh JW (2020). Absolute lymphocyte count predicts abscopal responses and outcomes in patients receiving combined immunotherapy and radiation therapy: analysis of 3 phase 1/2 trials. Int J Radiat Oncol Biol Phys.

[64] Herrera FG, Romero P, Coukos G (2022). Lighting up the tumor fire with low-dose irradiation. Trends Immunol.

[65] Menon H, Chen D, Ramapriyan R, Verma V, Barsoumian HB, Cushman TR (2019). Influence of low-dose radiation on abscopal responses in patients receiving high-dose radiation and immunotherapy. J Immunother Cancer.

[66] Ochoa de Olza M, Bourhis J, Irving M, Coukos G, Herrera FG. High versus low dose irradiation for tumor immune reprogramming. Curr Opin Biotechnol. 2020; 65:268–83. 10.1016/j.copbio.2020.08.001 32882511

[67] Welsh JW, Tang C, de Groot P, Naing A, Hess KR, Heymach JV (2019). Phase II trial of Ipilimumab with stereotactic radiation therapy for metastatic disease: outcomes, toxicities, and low-dose radiation-related abscopal responses. Cancer Immunol Res.

[68] Patel RR, He K, Barsoumian HB, Chang JY, Tang C, Verma V (2021). High-dose irradiation in combination with non-ablative low-dose radiation to treat metastatic disease after progression on immunotherapy: Results of a phase II trial. Radiother Oncol.

[69] Buchwald ZS, Wynne J, Nasti TH, Zhu S, Mourad WF, Yan W (2018). Radiation, immune checkpoint blockade and the abscopal effect: a critical review on timing, dose and fractionation. Front Oncol.

[70] Antonia SJ, Villegas A, Daniel D, Vicente D, Murakami S, Hui R (2018). Overall survival with durvalumab after chemoradiotherapy in stage III NSCLC. N Engl J Med.

[71] Woody S, Hegde A, Arastu H, Peach MS, Sharma N, Walker P (2022). Survival is worse in patients completing immunotherapy prior to SBRT/SRS compared to those receiving it concurrently or after. Front Oncol.

[72] Bauml JM, Mick R, Ciunci C, Aggarwal C, Davis C, Evans T (2019). Pembrolizumab after completion of locally ablative therapy for oligometastatic non-small cell lung cancer: a phase 2 trial. JAMA Oncol.

[73] Jabbour SK, Lee KH, Frost N, Breder V, Kowalski DM, Pollock T (2021). Pembrolizumab plus concurrent chemoradiation therapy in patients with unresectable, locally advanced, stage III non-small cell lung cancer: the phase 2 KEYNOTE-799 nonrandomized trial. JAMA Oncol.

[74] Twyman-Saint Victor C, Rech AJ, Maity A, Rengan R, Pauken KE, Stelekati E (2015). Radiation and dual checkpoint blockade activate non-redundant immune mechanisms in cancer. Nature.

[75] Young KH, Baird JR, Savage T, Cottam B, Friedman D, Bambina S (2016). Optimizing timing of immunotherapy improves control of tumors by hypofractionated radiation therapy. PLoS ONE.

[76] Ahn E, Araki K, Hashimoto M, Li W, Riley JL, Cheung J (2018). Role of PD-1 during effector CD8 T cell differentiation. Proc Natl Acad Sci U S A.

[77] Buchbinder EI, Desai A (2016). CTLA-4 and PD-1 pathways: similarities, differences, and implications of their inhibition. Am J Clin Oncol.

[78] Luchini C, Bibeau F, Ligtenberg MJL, Singh N, Nottegar A, Bosse T (2019). ESMO recommendations on microsatellite instability testing for immunotherapy in cancer, and its relationship with PD-1/PD-L1 expression and tumour mutational burden: a systematic review-based approach. Ann Oncol.

[79] Schoenfeld AJ, Rizvi H, Bandlamudi C, Sauter JL, Travis WD, Rekhtman N (2020). Clinical and molecular correlates of PD-L1 expression in patients with lung adenocarcinomas. Ann Oncol.

[80] Reck M, Rodriguez-Abreu D, Robinson AG, Hui R, Csoszi T, Fulop A (2016). Pembrolizumab versus chemotherapy for PD-L1-positive non-small-cell lung cancer. N Engl J Med.

[81] Bang A, Schoenfeld JD, Sun AY (2019). PACIFIC: shifting tides in the treatment of locally advanced non-small cell lung cancer. Transl Lung Cancer Res.

[82] Lu S, Stein JE, Rimm DL, Wang DW, Bell JM, Johnson DB (2019). Comparison of biomarker modalities for predicting response to PD-1/PD-L1 checkpoint blockade: a systematic review and meta-analysis. JAMA Oncol.

[83] Chan TA, Yarchoan M, Jaffee E, Swanton C, Quezada SA, Stenzinger A (2019). Development of tumor mutation burden as an immunotherapy biomarker: utility for the oncology clinic. Ann Oncol.

[84] Ettinger DS, Wood DE, Aisner DL, Akerley W, Bauman JR, Bharat A et al. Non-small cell lung cancer, version 3.2022, NCCN clinical practice guidelines in oncology. J Natl Compr Canc Netw. 2022; 20(5):497–530. 10.6004/jnccn.2022.0025 35545176

[85] Xia L, Liu Y, Wang Y (2019). PD-1/PD-L1 blockade therapy in advanced non-small-cell lung cancer: current status and future directions. Oncologist.

[86] Asaoka Y, Ijichi H, Koike K (2015). PD-1 blockade in tumors with mismatch-repair deficiency. N Engl J Med.

[87] Liu SY, Wu YL (2019). Biomarker for personalized immunotherapy. Transl Lung Cancer Res.

[88] Havel JJ, Chowell D, Chan TA (2019). The evolving landscape of biomarkers for checkpoint inhibitor immunotherapy. Nat Rev Cancer.

[89] Sun R, Limkin EJ, Vakalopoulou M, Dercle L, Champiat S, Han SR (2018). A radiomics approach to assess tumour-infiltrating CD8 cells and response to anti-PD-1 or anti-PD-L1 immunotherapy: an imaging biomarker, retrospective multicohort study. Lancet Oncol.

[90] Luo H, Ge H, Cui Y, Zhang J, Fan R, Zheng A (2018). Systemic inflammation biomarkers predict survival in patients of early stage non-small cell lung cancer treated with stereotactic ablative radiotherapy—a single center experience. J Cancer.

[91] Chen D, Patel RR, Verma V, Ramapriyan R, Barsoumian HB, Cortez MA (2020). Interaction between lymphopenia, radiotherapy technique, dosimetry, and survival outcomes in lung cancer patients receiving combined immunotherapy and radiotherapy. Radiother Oncol.

[92] Agrawal V, Benjamin KT, Ko EC (2020). Radiotherapy and immunotherapy combinations for lung cancer. Curr Oncol Rep.

[93] Brooks ED, Chang JY (2019). Time to abandon single-site irradiation for inducing abscopal effects. Nat Rev Clin Oncol.

[94] Luke JJ, Lemons JM, Karrison TG, Pitroda SP, Melotek JM, Zha Y (2018). Safety and clinical activity of pembrolizumab and multisite stereotactic body radiotherapy in patients with advanced solid tumors. J Clin Oncol.

